# Isolation and Characterization of Cellulose Nanocrystals from Rejected Fibers Originated in the Kraft Pulping Process

**DOI:** 10.3390/polym10101145

**Published:** 2018-10-14

**Authors:** María Graciela Aguayo, Arturo Fernández Pérez, Guillermo Reyes, Claudia Oviedo, William Gacitúa, Raúl Gonzalez, Omar Uyarte

**Affiliations:** 1 Centro de Biomateriales y Nanotecnología, Universidad del Bío-Bío, Concepción C.P. 4081112, Chile; wgacitua@ubiobio.cl; 2 Nanomateriales y Catálisis para Procesos Sustentables (NanoCatpPS), Depto. Ingeniería en Maderas Universidad del Bío-Bío, Concepción C.P. 4081112, Chile; 3 Facultad de Ingeniería, Depto. Ingeniería en Maderas, Universidad del Bío-Bío, Concepción C.P. 4081112, Chile; greyes@ubiobio.cl; 4 Facultad de Ciencias, Depto. Física, Universidad del Bío-Bío, Concepción C.P. 4081112, Chile; arturofe@ubiobio.cl; 5 Facultad de Ciencias, Depto. Química, Universidad del Bío-Bío, Concepción C.P. 4081112, Chile; coviedo@ubiobio.cl; 6 Centro Investigación y Desarrollo, CMPC Celulosa, Nacimiento C.P. 4550000, Chile; rgonzalez@celulosa.cmpc.cl (R.G.); omar.uyarte@cmpc.cl (O.U.)

**Keywords:** reject fibers, cellulose nanocrystals, acid hydrolysis, kraft process

## Abstract

In the final process of the bleached kraft pulp there are some cellulose fibers that are separated from the main fibers stream; these fibers are rejected and considered as a low quality fibers, these fibers are known as rejected fiber (RF). In the present work the potential use of these fibers for Cellulose Nanocrystals (CNCs) synthesis was studied. The physical and chemical properties of synthesized CNCs were characterized through different techniques such as Atomic Force Microscopy (AFM), Scanning Electron Microscopy (SEM), X-Ray Diffraction (XRD), Fourier-Transform Infrared Spectroscopy (FTIR), and Thermogravimetric Analysis (TGA). Results demonstrate the feasibility of CNCs synthesis with a yield of 28.1% and 36.9%, and crystallinity of 73.5% and 82.7%. Finally, the morphology and synthesis conditions suggest that this industrial reject fiber (RF) could be used as a source for the CNCs production, thus adding value to the kraft process and opening new possibilities for innovation in the pulp industry.

## 1. Introduction

The main process for the production of cellulose pulp worldwide is the kraft process. Only in 2016, approximately 138 million Air Dry ton (ADt, cellulose pulp with 10% of wet) were produced using this method, of which 26 million were produced in South America [1]. During the kraft process, various wastes are obtained; these can be considered as by-products according to their later utility. Among these we can mention (A) Lignin, used mainly as fuel for electricity, and also as a source of low content urea-formaldehyde adhesive resins [2], and of carbon fiber [3]; (B) Tall oil, obtained from coniferous extractives, that can be used to obtain phytosterols, as well as distilled tall oil in different fractions, such as Tora, Tofa, and Pitch [4]; (C) Effluent sludge that can be burned to obtain concentrated energy through pyrolysis [5] or through hydrothermal carbonization processes [6].

In the final process of depuration of the bleached pulp there is a daily loss of some cellulose fibers, which can be recovered by filtering and pressing processes. This fiber is known as secondary fiber or rejected fiber (RF). In terms of quality, the high content of impurities and vessels make RF an inferior product that does not meet market standards. However, its physical and mechanical properties are similar to bleached pulp for exportation. Currently, RF reaches an approximate commercial value of US$100/wet ton, and is used as raw materials for other industries (mainly in cardboard business), becoming an example of circular economy [7].

RF presents a high economical potential that has not been evaluated so far. If we consider that the cost of delignification is already assumed by the kraft industry, this fiber could be used as a raw material for cellulose nanofibrils (CNFs) or cellulose nanocrystals (CNCs) production. CNCs have received great attention in the global scientific community for their unique mechanical and optical properties [8,9,10,11,12]. CNCs are commonly produced by acid hydrolysis of cellulose fibers [13,14,15,16,17,18,19,20]. Sulfuric acid is one of the most used inorganic acids for CNCs production, providing good dispersion of the CNC in suspension after hydrolysis, since the sulfate ion exhibits negative charges and can cause electrostatic repulsion between among the CNCs molecules [21,22]. However, the high stability this CNCs compromises the thermal stability of the nanoparticles [23]. The morphology and crystallinity of CNCs depends on the origin of the cellulosic substrate and hydrolysis conditions. Indeed, it has been previously reported that higher temperatures in a short reaction time yield CNCs with a higher crystallinity index than those obtained under mild reaction conditions [24]. On the other hand, the CNCs crystalline planes can be ordered in a different way depending on the synthesis conditions [25], therefore having a variation between the distances of the crystalline planes, which could provide useful information about the stress and deformation present within the CNCs, and finally having an effect on physical properties at macroscopic level.

In resume the goal of this work was to analyze the potential use of a kraft industry by-product, the so called rejected fiber (RF) as a raw material for the production of cellulose nanocrystals (CNCs) detailed information and discussion on the CNCs synthesized is presented.

## 2. Materials and Methods

### 2.1. Materials

**Figure 1:**
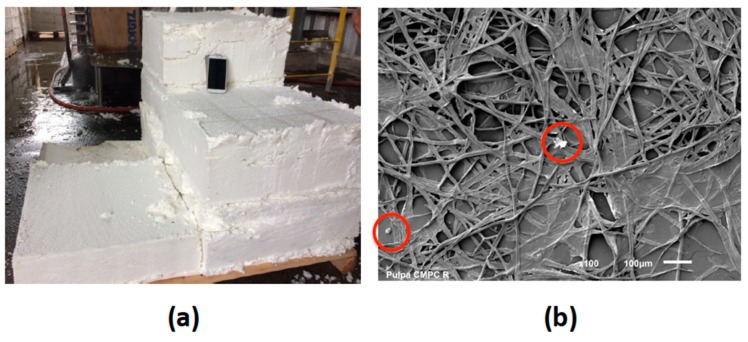
(**a**) Digital photograph of reject fibers collected from fibers purification system; (**b**) SEM image of reject fibers highlight in circles it is observed impurities that these fibers present (bar scale: 100 μm).

RF samples were obtained from Santa Fe Kraft pulp mill (CMPC Celulosa S.A., Nacimiento, Chile). This pulp mill line uses eucalyptus mixes (*Eucalyptus globulus* and *Eucalyptus nitens*) as a raw material. The cooking process corresponds to Low Solid technology and the sequence of bleaching process is D_0_E_OP_D_1_. After the bleaching and washing processes the fibers pass through a serial of depuration steps to remove impurities by physical (decanting) and mechanical (mesh) processes. Further details are not provided due to industrial privacy policies. The fibers used in this work were collected from this fifth stage of the purification system (Figure 1). The samples were disintegrated into a fiber suspension at 5% solids concentration. Wet pulps were then vacuum, dewatered, and air dried to 8% moisture. The chemical and physical characteristics of RF provided are shown in Table 1.

### 2.2. Preparation of CNCs

CNCs from RF were prepared by sulfuric acid hydrolysis [17,28]. According to preliminary test for RF and studies reported to obtain CNCs, two conditions of hydrolysis were evaluated. Table 2 shows treatment conditions: sulfuric acid concentration, reaction temperature, and reaction time utilized. Five grams of RF sample were added to 200 mL of the acid solution, the resulting solutions were mechanically stirred during the reaction. The reaction was stopped with cold distilled water and the reaction products were washed three consecutive times by centrifugation (10 min, 12,000 rpm). The resulting suspensions were dialyzed against distilled water to constant pH. Finally, the samples were sonicated in an iced-water bath for 30 min and centrifuged at 9000 rpm for 5 min. Thus, the resulting supernatants containing the isolated CNCs were kept at 4 °C for further analysis. Their respective residues were cellulose fibers that were not completely hydrolyzed, called cellulosic solid residues (CSRs), that were dry weighed for yield estimations as described in Section 2.3.

### 2.3. CNCs and CSR Yields

Chemical oxygen demand (COD) measurements were used to determine CNCs yield according to procedure by Wang et al. [17]. The method of analysis is done by closed reflux and the samples are subsequently analyzed colorimetrically. First, the linearity and repeatability of a calibration curve of a potassium hydrogen phthalate solution was verified in the COD range of 25–1500 mg L^−1^ O_2_. Avicel^®^ samples were used for calibration and further calculations. A proper aliquot was taken and added in the COD reaction tubes (Spectroquant^®^ COD cell test). The tubes were thoroughly mixed in a vortex mixer and incubated for 2 h at 148 °C in a Spectroquant^®^ 320 Thermoreactor (MERCK, Darmstadt, Germany). Once the samples were cooled down to room temperature, the absorbance at 600 nm was measured on a spectrophotometer Shimadzu UV-vis 1603, and the COD was determined by interpolation with avicel calibration curve samples. On the other hand, the separated CSRs were measured using the gravimetric method that consists in overnight oven drying at 105 °C, finally the dry weight was used to calculate the CSR yield for each sample.

### 2.4. CNCs and CSR Images

After hydrolysis, the CSR products were examined using a scanning electron microscope (SEM, JEOL JSM6610LV, JEOL Ltd., Tokyo, Japan). Initially, samples were coated with gold by dc-sputtering technique using a Denton Vacuum Desk V-Sputter/etch unit, with 20 mA of current for 30 s. Then, the samples were observed with an acceleration voltage of 10 kV.

Surface morphology for CNCs samples was examined using an atomic force microscope (NaioAFM, Nanosurf, Liestal, Switzerland). The equipment was operated in phase contrast mode using PPP-FMAuD Gold Coated Force Modulation AFM Probes (Nanosensors, Neuchâtel, Switzerland) at a resonance frequency of 75 kHz, spring constant of 2.8 N m^−1^, and tip radius of about 7 nm. For this purpose, the original CNCs solution was diluted fivefold with deionized water and sonicated for 30 min. Then, one drop of the diluted solution was deposited on a regular microscope slide and put it in a vacuum drying oven al 60 °C and 0.5 bar of air pressure for 30 min. After this procedure the samples are ready for AFM data acquisition, where the time of each essay was 310 ms per line and each micrograph has 1024 lines of resolution. From the AFM images obtained, 150 diameter measurements of the CNCs were taken, and average values were determined by using the ImageJ software (Fiji distribution, open-source) [20].

### 2.5. Chemical Characterizations of CNCs

#### 2.5.1. Sulfur Content Analysis

The sulfur content of each CNC was analyzed using an ICP-AES (Optima 800, PerkinElmer, Waltham, MA, USA) according to Chen et al. [28]. An aliquot of 5 mL of CNC suspension was transferred to a Teflon digestion flask and digested at 150 °C for 30 min in a microwave (Ethos One, Milestone, Sorisole, Italy) with 5 mL of 70% HNO_3_ before ICP-AES analysis. The time ramp digestion at 150 °C was approximately 40 min.

#### 2.5.2. Fourier Transform Infrared (FTIR) Spectroscopy

FTIR spectroscopy was used to examine the changes in the functional groups of CNCs obtained. The FTIR spectra were collected by using a Nicolet 380 FT-IR Spectrometer (Thermo Fisher Scientific, Hampton, NH, USA) in the transmittance mode. Five mg of powder samples of RF and CNC were dispersed in a matrix of KBr to be mixed and pressed into a pellet. The samples were analyzed in a spectral region between 4000 and 400 cm^−1^ with a 2 cm^−1^ resolution, while the obtained spectra were averaged over 32 scans.

#### 2.5.3. X-ray Diffraction (XRD) Analysis

The X-ray diffraction (XRD) analysis was performed to determine the crystallinity of the samples from by-products of pulp mill and CNCs obtained. The samples were examined using WAXS (wide angle X-ray scattering) equipment, in the X-ray diffractometer Rigaku Smartlab^®^. Approximately 0.05 g was used for each experiment on the sample holder. Angular scanning was conducted from 5° to 50° with 5° min^−1^ with Cu Kα radiation (λ = 0.154059 nm), and the generator was working at 45 kV and 200 mA. The analysis of cellulosic material was performed according to reported crystallographic data by Ford et al. [29] and Nam et al. [30], using this information as an initial step to start and optimize the deconvolution of X-ray diffraction patterns.

The crystallite size *τ* (nm), perpendicular to the lattice plane (002) cellulose I was calculated in Equation (1) by the Scherrer equation [31], where *K* is the Scherrer constant (0.94), *λ* is the wavelength of the X-ray radiation (0.154 nm), and *β* is the full width at half maximum of the diffraction peak (in radians) and θ is the diffraction angle of the peak.

(1) $$\;\tau =\frac{K\lambda }{\beta cos\theta }\;$$

Also, the fractional variation in the plane spacing Δ*d*/*d* for the (002) planes was calculated following the Equation (2), according to Cullity and Stock [32].

(2) $$\;\left|\frac{\Delta d}{d}\right|=\frac{\beta }{2tan\theta }\;$$

The Segal *CI* was calculated according to Equation (3), where it is the total intensity of the (002) peak for cellulose I, and *Ia* is the amorphous intensity [33].

(3) $$\;CI=\frac{I_{t}-I_{a}}{I_{t}}\times 100\;$$

#### 2.5.4. Thermogravimetric Analysis (TGA)

The thermogravimetric analysis of sample CNCs and RF was carried out on a TGA Q50 (TA Instruments, New Castle, DE, USA) under nitrogen atmosphere, with a gas flow of 50 mL min^−1^ from 25 to 600 °C at a heating rate of 10 °C min^−1^. The weight-loss rate was obtained from derivate thermogravimetric data. For this analysis, about 2.0 mg of powder sample RF and CNCs were used.

## 3. Results

### 3.1. Morphological Analysis

**Figure 2:**
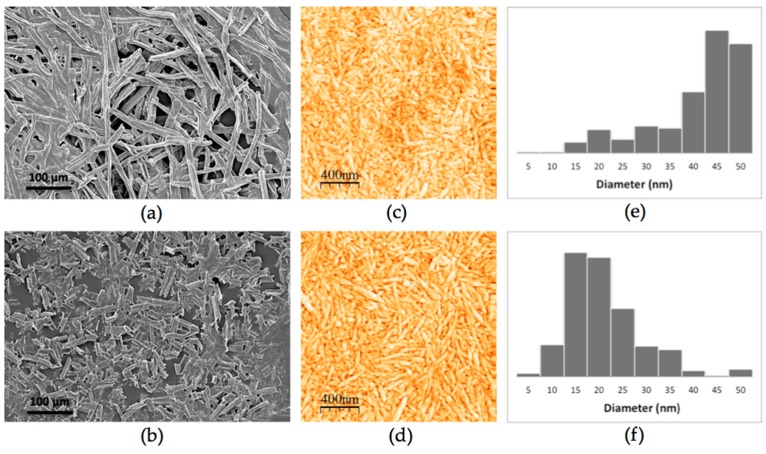
(**a**,**b**) corresponds to SEM images of cellulosic solid residue (CSR-1 and CSR-2, respectively); (**c**,**d**) correspond to AFM images of cellulose nanocrystals obtained (CNC-1 and CNC-2, respectively); (**e**,**f**) correspond to the size distribution of the CNCs obtained (CNC-1 and CNC-2, respectively).

Morphology and size distribution of CNCs and its corresponding cellulosic solid residue (CSR) synthesized are shown in Figure 2. Figure 2a,b corresponds to images SEM of the RF that were not completely hydrolyzed, thus generating CSR, derived from experiments 1 and 2 (CSR-1, CSR-2) respectively. Figure 2b, shows a higher fractionation of cellulose fibers for the case of CSR-2 compared to CSR-1 (Figure 2a), such difference on fractionation might be due to the different hydrolysis conditions, resulting in different CNCs size distributions. On the other hand, AFM images shown a bigger size distribution for CNCs obtained in CSR-1 compared to those in CSR-2 (Figure 2c,d). The CNCs obtained are morphologically similar to those reported from by Cranston and Gray [34], derived from cotton fibers, and to those, reported by Leung et al. [35], derived from Flax.

There are factors that can affect the CNCs production and their properties for instance; the cellulose source could impact in the size distribution and morphology of nanocrystals, besides other factors such as temperature and hydrolysis time [36]. The results shown that CNCs can be produced from RF for the experimental conditions evaluated, though differences in the size distribution of their diameters were found, CNC-1 presents a high percentage of nanoparticles with a diameter greater than 40 nm (Figure 2e) and CNC-2 presents a homogeneous distribution of nanoparticles with diameter values between 10 and 35 nm (Figure 2f).

### 3.2. Yields and Sulfur Content

The results of CNCs and CSR yields are shown in Table 3. Yields for CNC-1 and CNC-2 were 28.1% and 36.9% respectively (determined by COD). The production of CSR can be visually estimated from the precipitated cellulosic fibers in the post dialysis sample and quantified by the final solids remaining after overnight oven dry (105 °C). CNC-1 yield was 28.1%, which is less than the value reported by Wang et al. [17] that had a CNC yield of 39.5%, under the same conditions for high-quality bleached kraft pulp as a starting material. On the other hand, CNC-2 yield was similar to previous reports using an acid concentration of 64.8 wt % with CNC yield of 38%, using pure microcrystalline cellulose as the starting material [14].

Other parameter that has been considered of importance for CNCs acid hydrolysates is the sulfation [37], the use of sulfuric acid for hydrolysis induces grafting of negatively charged sulfate groups on the nanocrystals surface, thus contributing to the stabilization of colloidal suspensions by repulsive interparticle forces [38]. The sulfur content determined for CNC-1 and CNC-2 was of 8.4 and 12.4 mg g^−1^ respectively (Table 3), similar values to those reported in literature [14,17]. From Table 3, is possible to notice that the sulfur content in CNC-2 is higher than in CNC-1, this due to the fact that sulfation is affected mainly by high acid concentration. Nevertheless, it worth noticing that the presence of sulfate groups on the nanocrystals surface might be disadvantageous for some applications, since this favors its decomposition at various temperatures [20].

### 3.3. FTIR Analysis

**Figure 3:**
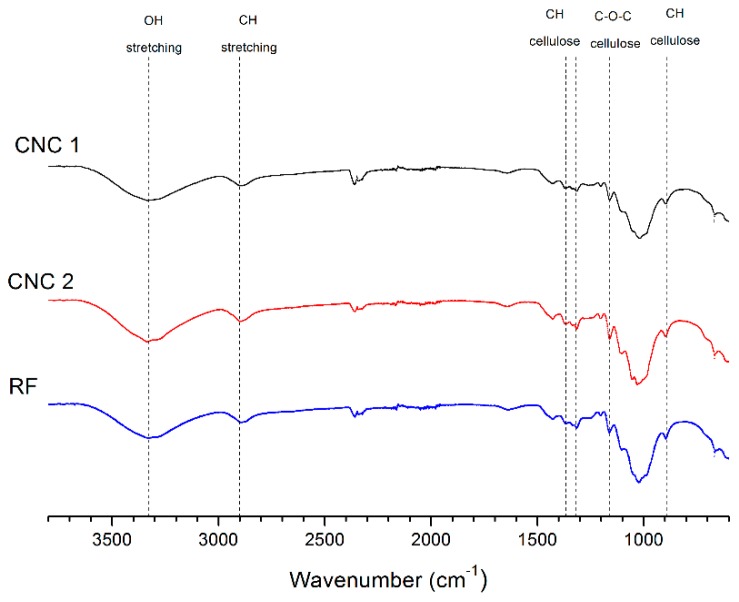
FTIR spectra for RF and CNCs obtained.

Following the chemical characterization, fourier-transform infrared spectroscopy (FTIR) was used to identify chemical group into the raw material (RF) and the mains samples CNC-1 and CNC-2, Figure 3 shows the FTIR spectra for those samples in the range of 600–3800 cm^−1^.

From Figure 3, it is possible to notice the following characteristics: the absorbance peaks in the 3400–3300 cm^−1^ regions are attributed to the stretching and bending vibrations of the OH groups of cellulose, respectively. The peaks around 2900–2800 cm^−1^ correspond to CH stretching [39]. The CH stretching region is little analyzed, because cellulose, hemicelluloses, and lignin have similar peak shapes in this band [40,41]. The band at 1642 cm^−1^ of de RF and CNCs obtained is associated to adsorbed water in cellulose [16,41,42] The peaks observed in the range of 1420–1430 cm^−1^ were attributed to the symmetric bending of CH_2_ [41] and were also related to cellulose I [41,42], the band at 1330–1380 cm^−1^ corresponded to the bending vibrations of the C–H and C–O groups of the polysaccharides [16,43]. The absorbance peaks observed in the 1161 cm^−1^ range were attributed to C–O–C asymmetric stretching vibrations associated with cellulose I and cellulose II [44]. Finally, the obtained spectra showed a slight increase in the peak intensities around 900 cm^−1^ which could be attributed to interactions between glycosidic linkages and glucose units of the cellulose [45]. Finally, the FTIR spectra of reject fiber and CNCs samples indicate that CNCs were successfully extracted from the hydrolysis treatment without any further degradation or secondary products formation. No significant changes were observed in the FTIR spectrum of the CNC obtained.

### 3.4. XRD Analysis of CNCs

**Figure 4:**
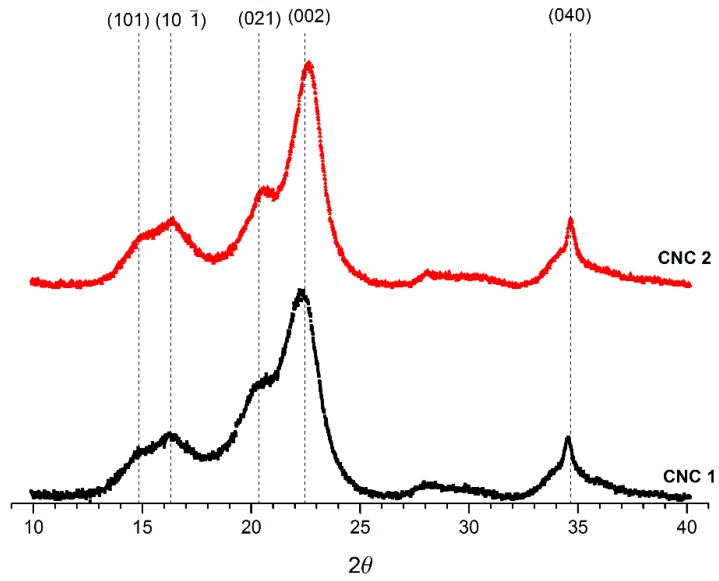
XRD diffractograms for CNC-1 and CNC-2.

The crystallinity of the CNCs obtained was investigated by XRD (Figure 4). The results indicated that the CNCs (1 and 2) correspond mainly to cellulose type I, with a high index of crystallinity, and both have the particularity that they show the peak around 20.5°. This is a characteristic for crystals with miller index (021) in cellulose type I polymorph, and this is particular because this peak does not always appear in all type I cellulose samples [27,28]. In general, the same type of crystalline planes observed for CNC-1 were observed for CNC-2 samples, with the difference that for CNC-1 samples, the peak with Miller indexes (101) and (10∑1) become smaller and the amount of cellulose in amorphous state become larger. This information is confirmed when crystallinity of the samples is calculated.

As can be observed in Table 4, crystallinity in CNC-2 samples is higher compares to those in CNC-2 samples. Besides, smaller crystallite size is found in CNC-1, with a high fractional variation in its interplanar distance, this last magnitude is used to analyze the strain and the microstresses between crystalline planes [32]. In this sense, we found that CNC-2 planes have less strain between them than CNC-1, and this could be related with a better crystal quality of CNC-2 in comparison with CNC-1. The fractional variation of the interplanar distance is a measurement for the dispersion of the crystalline planes values, within the crystallite; therefore, a greater value is associated with higher microstresses on the CNC [46]. This microstress could finally influence the physical properties of future materials or nanocomposite that can be developed using these CNCs as raw material.

### 3.5. Thermogravimetric Analysis

**Figure 5:**
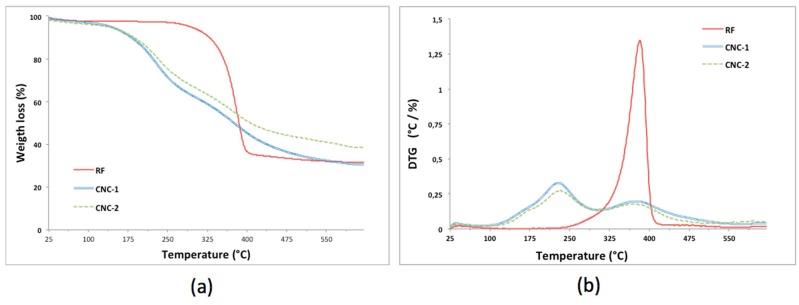
(**a**) TG and (**b**) DTG curves for the RF and CNCs obtained.

Thermal analysis of the CNC was investigated, and Figure 5 shows the TGA profiles for RF and CNCs samples. All the samples present similar thermal behavior in agreement with reported studies [16,20]. The weight loss below 140–180 °C could be attributed to water evaporation; additionally, two degradation stages were evident around 230 and 350 °C. These findings indicate that the thermal stability of cellulose nanocrystals prepared by sulfuric acid hydrolysis were lower than those for the RF sample, this due to the great number of free end chains in CNC surface derived from the small particle size, those end chains regularly start to decompose at low temperature [47].

It is well known that there is a “size effect” of the nanometersized crystallites in the in-plane direction on the thermal conduction, and that the cellulose crystallites transport heat more effectively with increasing cross-sectional area, being this a dominant factor with respect to the in-plane thermal conductivity [48]. This collected information agrees with the results of this study where CNC-2 exhibited higher crystanility and crystallite size, and lower interplaner distance compared to CNC-1 samples (see Table 4).

## 4. Conclusions

Cellulose nanocrystals (CNC) were synthesized from industrial secondary products (Rejected Fiber-RF) by acid hydrolysis with sulphuric acid. The yield obtained and the method for its measurement was discussed in order to present a consistent methodology for CNC production and a proper characterization of it. The results obtained reinforce the value of the degree of sulfation as a valuable indicator of the performance of NCC yield by sulphuric hydrolysis, and demonstrate the feasibility of obtaining thoroughly characterized CNCs of well-defined structure using a kraft industry by-products. The properties of the crystallites of the CNCs obtained reveal an important potential for the RF as a source for the industrial production of CNCs, offering a product of high crystallinity and of similar characteristics to those from sources of higher cost, such as pine or eucalyptus wood.

## Figures and Tables

**Table 1:** Chemical composition and physical properties of reject fibers (RF).

Properties	Value
Glucans * (%)	78.1 ± 1.3
Xylans * (%)	14.1 ± 0.3
Lignin * (%)	0.2 ± 0.1
Ash * (%)	1.7 ± 0.2
Fiber length ** (mm)	0.75
Fiber width ** (μm)	18.6
Vessels per 100,000 fibers **	409
Coarseness (mg 100 m^−1^) **	7.9

* Chemical composition were determined according to Ferraz et al. [26] and ASTM D1102-84 [27]. ** Physical properties were determined in Fiber Tester equipment (Lorentzen and Wettre, Stockholm, Sweden).

**Table 2:** Conditions of hydrolysis for obtain CNCs.

Sample	H_2_SO_4_ (%)	Reaction Time (min)	Reaction Temperature (°C)
CNC-1	60	102	55
CNC-2	64.8	10	40

**Table 3:** Yields of CNCs and CSRs and sulfur contents of CNCs.

Sample	CNC Yield ^a^ (%)	CSR Yield ^b^ (%)	CNC Sulfur Content (mg g^−1^)
CNC-1	28.1	45.8	8.4
CNC-2	36.9	41.3	12.4

^a^ yield by COD; ^b^ yield by drying.

**Table 4:** Crystallographic parameters for CNCs obtained.

Sample	$\frac{\Delta d}{d}\;$	*τ* (nm)	CI (%)
CNC-1	0.0782	4.778	73.5
CNC-2	0.0685	5.423	82.7

## References

[1] Food and Agriculture Organization of the United Nations–FAO (2016). Pulp and Paper Capacities.

[2] Siddiqui H. (2013). Production of Lignin-Based Phenolic Resins Using De-Polymerized Kraft Lignin and Process Optimization. Master’s Thesis.

[3] Mainka H., Hilfert L., Busse S., Edelmann F., Haak E., Herrmann A.S. (2015). Characterization of the major reactions during conversion of lignin to carbon fiber. J. Mater. Res. Technol..

[4] Diaz M.A.F., Rojas A.M., Hinojosa R.B.L., Schersl E.M. (2001). Process for Obtaining Unsaponifiable Compounds from Black-Liquor Soaps, Tall Oil and Their by-Products. U.S. Patent.

[5] Magalhães W.L.E., Jobb A.E., Ferreira C.A., da Silva H.D. (2008). Pyrolysis and combustion of pulp mill lime sludge. J. Anal. Appl. Pyrolysis.

[6] Monte M.C., Fuente E., Blanco A., Negro C. (2013). Waste management from pulp and paper production in the European Union. Waste Manag..

[7] Murray A., Skene K., Haynes K. (2017). The Circular Economy: An Interdisciplinary Exploration of the Concept and Application in a Global Context. J. Bus. Ethics.

[8] Dong X.M., Gray D.G. (1997). Effect of counterions on ordered phase formation in suspensions of charged rodlike cellulose crystallites. Langmuir.

[9] Eichhorn S.J., Dufresne A., Aranguren M., Marcovich N.E., Capadona J.R., Rowan S.J., Weder C., Thielemans W., Roman M., Renneckar S. (2010). Review: Current international research into cellulose nanofibres and nanocomposites. J. Mater. Sci..

[10] Habibi Y., Lucia L.A., Rojas O.J. (2010). Cellulose nanocrystals: Chemistry, self-assembly and applications. Chem. Rev..

[11] Klemm D., Kramer F., Moritz S., Lindström T., Ankerfors M., Gray D., Dorris A. (2011). Nanocelluloses: A new family of nature-based materials. Angew. Chem. Int. Ed..

[12] Kaushik M., Moores A. (2016). Review: Nanocelluloses as versatile supports for metal nanoparticles and their applications in catalysis. Green Chem..

[13] Beck-Candanedo S., Roman M., Gray D.G. (2005). Effect of reaction conditions on the properties and behavior of wood cellulose nanocrystal suspension. Biomacromolecules.

[14] Bondenson D., Mathew A., Oksman K. (2006). Optimization of the isolation of manocrystals from microcrystalline cellulose by acid hydrolysis. Cellulose.

[15] Chieng B.W., Lee S.H., Ibrahim N.A., Then Y.Y., Loo Y.Y. (2017). Isolation and Characterization of Cellulose Nanocrystals from Oil Palm Mesocarp Fiber. Polymers.

[16] Kargarzadeh H., Ahmad I., Abdullah I., Dufresne A., Zainudin S.Y., Sheltami R.M. (2012). Effects of hydrolysis conditions on the morphology, crystallinity, and thermal stability of cellulose nanocrystals extracted from kenaf bast fibers. Cellulose.

[17] Wang Q.Q., Zhu J.Y., Reiner R.S., Verrill S.P., Baxa U., McNeil S.E. (2012). Approaching zero cellulose loss in cellulose nanocrystal (CNC) production: Recovery and characterization of cellulosic solid residues (CSR) and CNC. Cellulose.

[18] Fortunati E., Puglia D., Monti M., Peponi L., Santulli C., Kenny J.M., Torre L. (2013). Extraction of cellulose nanocrystals from *Phormium tenax* fibres. J. Polym. Environ..

[19] Wang Q., Zhao X., Zhu J.Y. (2014). Kinetics of strong acid hydrolysis of a bleached kraft pulp for producing cellulose nanocrystals (CNCs). Ind. Eng. Chem. Res..

[20] De Oliveira F.B., Bras J., Pimenta M.T.B., da Silva Curvelo A.A., Belgacem M.N. (2016). Production of cellulose nanocrystals from sugarcane bagasse fibers and pith. Ind. Crops Prod..

[21] Pereira A.L.S., do Nascimento D.M., Morais J.P.S., Vasconcelos N.F., Feitosa J.P., Brígida A.I.S., Rosa M.D.F. (2014). Improvement of polyvinyl alcohol properties by adding nanocrystalline cellulose isolated from banana pseudostems. Carbohydr. Polym..

[22] Wang Z., Yao Z., Zhou J., Zhang Y. (2017). Reuse of waste cotton cloth for the extraction of cellulose nanocrystals. Carbohydr. Polym..

[23] Roman M., Winter W.T. (2004). Effect of Sulfate Groups from Sulfuric Acid Hydrolysis on the Thermal Degradation Behavior of Bacterial Cellulose. Biomacromolecules.

[24] Teodoro K.B.R., Teixeira E.M., Corrêa A.C., de Campos A., Marconcini J.M., Mattoso L.H.C. (2011). Whiskers de fibra de sisal obtidos sob diferentes condições de hidrólise ácida: Efeito do tempo e da temperatura de extração. Polím. Ciênc. Tecnol..

[25] Ornaghi H.L., Poletto M.P., Zattera A.J., Amico S.C. (2014). Correlation of the thermal stability and the decomposition kinetics of six different vegetal fibers. Cellulose.

[26] Ferraz A., Mendonca R., Guerra A., Ruiz J., Rodríguez J., Baeza J., Freer J. (2005). Near Infrared Spectra and chemical characteristics of *Pinus taeda* (Loblolly Pine) wood chips biotreated by the white rot fungus *Ceriporiopsis subvermispora*. J. Wood Chem. Technol..

[27] ASTM D1102-84 (2013). Standart Test Method for Ash in Wood.

[28] Chen L., Wang Q., Hirth K., Baez C., Agarwal U.P., Zhu J.Y. (2015). Tailoring the yield and characteristics of wood cellulose nanocrystals (CNC) using concentrated acid hydrolysis. Cellulose.

[29] Ford E.N.J., Mendon S.K., Thames S.F., Rawlins J.W. (2010). X-ray diffraction of cotton treated with neutralized vegetable oil-based macromolecular crosslinkers. J. Eng. Fibres Fabr..

[30] Nam S., French A.D., Condon B.D., Concha M. (2016). Segal crystallinity index revisited by the simulation of X-ray diffraction patterns of cotton cellulose Iβ and cellulose II. Carbohydr. Polym..

[31] Scherrer P. (1918). Bestimmung der Größe und der inneren Struktur von Kolloidteilchen mittels Röntgenstrahlen. Nachrichten von der Gesellschaft der Wissenschaften zu Göttingen, Mathematisch-Physikalische Klasse.

[32] Cullity B.D., Stock S.R. (2001). Elements of X-ray Diffraction.

[33] Segal L., Creely J.J., Martin A.E., Conrad C.M. (1959). An empirical method for estimating the degree of crystallinity of native cellulose using the X-ray diffractometer. Text. Res. J..

[34] Cranston E.D., Gray D.G. (2006). Morphological and optical characterization of polyelectrolyte multilayers incorporating nanocrystalline cellulose. Biomacromolecules.

[35] Leung A.C., Hrapovic S., Lam E., Liu Y., Male K.B., Mahmoud K.A., Luong J.H. (2011). Characteristics and properties of carboxylated cellulose nanocrystals prepared from a novel one-step procedure. Small.

[36] Rahimi M., Behrooz R. (2011). Effect of cellulose characteristic and hydrolyze conditions on morphology and size of nanocrystal cellulose extracted from wheat straw. Int. J. Polym. Mater..

[37] Hamad W.Y., Zhu J.Y., Zhang X., Pan X.J. (2011). Development and properties of nanocrystalline cellulose (NCC). Sustainable Production of Fuels, Chemicals, and Fibers from Forest Biomass.

[38] Dong X.M., Revol J.F., Gray D.G. (1998). Effect of microcrystallite preparation conditions on the formation of colloid crystals of cellulose. Cellulose.

[39] Sain M., Panthapulakkal S. (2006). Bioprocess preparation of wheat straw fibres and their characterization. Ind. Crops Prod..

[40] Barnette A.L., Lee C., Bradley L.C., Schreiner E.P., Park Y.B., Shin H., Cosgrove D.J., Park S., Kim S.H. (2012). Quantification of crystalline cellulose in lignocellulosic biomass using sum frequency generation (SFG) vibration spectroscopy and comparison with other analytical methods. Carbohydr. Polym..

[41] Carrillo I., Mendonça R.T., Ago M., Rojas O.J. (2018). Comparative study of cellulosic components isolated from different Eucalyptus species. Cellulose.

[42] Tsuboi M. (1957). Infrared spectrum and crystal structure of cellulose. J. Polym. Sci..

[43] Jonoobi M., Harun J., Shakeri A., Misra M., Oksman K. (2009). Chemical composition, crystallinity, and thermal degradation of bleached and unbleached kenaf bast (*Hibiscus cannabinus*) pulp and nanofibers. BioResources.

[44] Alemdar A., Sain M. (2008). Isolation and characterization of nanofibres from agricultural residues-wheat straw and soy hulls. Bioresour. Technol..

[45] Li R., Fei J., Cai Y., Li Y., Feng J., Yao J. (2009). Cellulose whiskers extracted from mulberry: A novel biomass production. Carbohydr. Polym..

[46] Gusev A.I., Rempel A.A. (2004). Nanocrystalline Materials.

[47] Wang N., Ding E., Cheng R. (2007). Thermal degradation behaviors of spherical cellulose nanocrystals with sulfate groups. Polymer.

[48] Uetani K., Okada T., Oyama H.T. (2015). Crystallite size effect on thermal conductive properties of nonwoven nanocellulose sheets. Biomacromolecules.

